# Recent trends in smartphone-based detection for biomedical applications: a review

**DOI:** 10.1007/s00216-021-03184-z

**Published:** 2021-02-15

**Authors:** Soumyabrata Banik, Sindhoora Kaniyala Melanthota, Arbaaz, Joel Markus Vaz, Vishak Madhwaraj Kadambalithaya, Iftak Hussain, Sibasish Dutta, Nirmal Mazumder

**Affiliations:** 1 Department of Biophysics, Manipal School of Life Sciences, Manipal Academy of Higher Education, Manipal, Karnataka 576104 India; 2 Department of Biotechnology, Manipal Institute of Technology, Manipal Academy of Higher Education, Manipal, Karnataka 576104 India; 3 Center for Healthcare Entrepreneurship, Indian Institute of Technology, Hyderabad, Telangana 502285 India; 4 Department of Physics, Pandit Deendayal Upadhyaya Adarsha Mahavidyalaya (PDUAM), Eraligool, Karimganj, Assam 788723 India

**Keywords:** Smartphone, Optical microscopy, Fluorescence imaging, Immunoassay, Diagnostics, Deep learning

## Abstract

Smartphone-based imaging devices (SIDs) have shown to be versatile and have a wide range of biomedical applications. With the increasing demand for high-quality medical services, technological interventions such as portable devices that can be used in remote and resource-less conditions and have an impact on quantity and quality of care. Additionally, smartphone-based devices have shown their application in the field of teleimaging, food technology, education, etc. Depending on the application and imaging capability required, the optical arrangement of the SID varies which enables them to be used in multiple setups like bright-field, fluorescence, dark-field, and multiple arrays with certain changes in their optics and illumination. This comprehensive review discusses the numerous applications and development of SIDs towards histopathological examination, detection of bacteria and viruses, food technology, and routine diagnosis. Smartphone-based devices are complemented with deep learning methods to further increase the efficiency of the devices.

**Figure:**
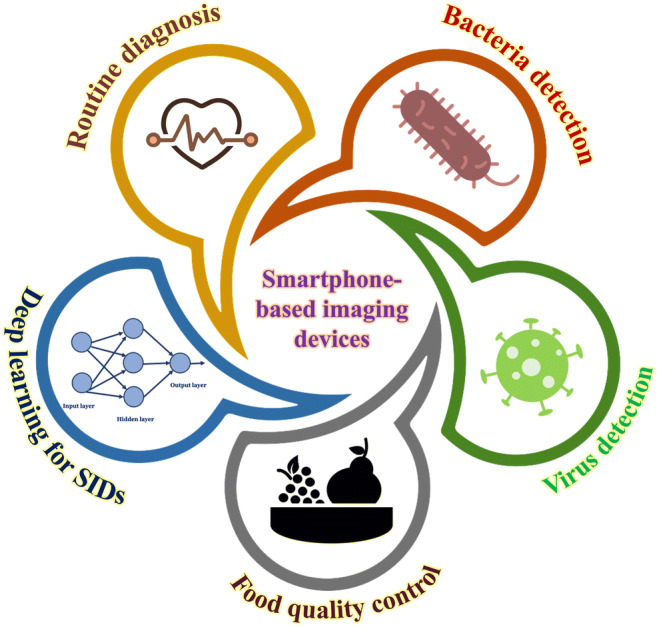
Graphical Abstract

Graphical Abstract

## Introduction

The microscope since its invention has been an important imaging tool in understanding modalities of the underlying micro-world. Microscopes have constantly evolved in terms of the resolution providing high sensitivity for pathogen detection and disease diagnosis. These high-end microscopes are often not easily available to the masses in remote areas due to lack of technical facilities, thus delaying in-time diagnosis causing fatalities in resource-less areas [1]. Smartphone-based imaging devices (SIDs) are platforms that utilize the imaging capability of a smartphone and is used for applications other than conventional photography. They have shown to be utilized as microscopic devices, analytical detection and sensing devices, devices to monitor pollution and contaminants, as well as devices for educational purposes. SIDs are advantageous over the conventional optical microscope in areas with limited manpower and where the requirement of rapid diagnosis is high. SIDs are portable with either add-on attachment or modification of the built-in camera setup for advanced imaging. Other benefits could be diagnosis confirmation, sharing of knowledge, and rapid data sharing for faster analysis. This makes the whole diagnostic system affordable and helps in reaching out to a larger section of the society [2]. The modern smartphone with improved hardware and software integration can be used as a stand-alone platform in terms of inbuilt source, detector, and the computational platform, and has been continuously exploited to develop autonomous mobile sensing devices which find its applicability for various analytical applications [3–5]. The rapid development of the smartphone has made it a foremost choice in the area of affordable biomedical services. Different optical improvement and technique incorporation have been used to convert the smartphone into a laboratory-grade microscopic device to carry out various bioanalytical analysis. Using a single 1-mm ball lens and light-emitting diode (LED) configuration, Zhu et al. have demonstrated the usability of the smartphone as a cost-effective imaging tool for rapid blood analysis [6]. D’Ambrosio et al. have demonstrated the first video microscopy platform in a smartphone and its usability for the detection and evaluation of blood-borne filarial parasites [7]. Using the traditional microscopic technique, several research groups have demonstrated smartphone-based fluorescence imaging platforms with enhance sensitivity and specificity [8, 9]. Wei et al. have demonstrated a smartphone-based fluorescent imaging platform to image individual fluorescent nanoparticles having a diameter of approximately 100 nm [10]. In the digital holographic imaging scheme, smartphone-based imaging can be possible without any external lens configuration. In this scheme, the shadow of the sample is allowed to fall on the bare complementary metal-oxide-semiconductor (CMOS) chip of the smartphone camera, and using rigorous image processing algorithms, the image can be reconstructed from the digital hologram created on the sensor. Lee et al. have demonstrated a smartphone-based lens-less microscope utilizing ambient illuminance as a light source [11]. Similarly, Roy et al. have demonstrated the lens-free shadow imaging technique in a smartphone platform for counting blood cells [12], whereas Ozcan et al. have used a fluorescent optofluidic-based imaging platform to measure the density of white blood cells [13].

There are several scientific articles on the development of different SIDs as well as elucidating their applications. However, it is often difficult for new readers to find a comprehensive review to understand the basic principles of such devices and a wide range of multidisciplinary biomedical relevance. In this review article, we discuss various applications of SIDs in histopathology, pathogen detection, food technology, diagnosis, machine learning integrated to a smartphone, and specialized 3D-printed optomechanical attachments containing sensors for particular examinations. Table 1 further summarizes various biomedical applications of SIDs along with the advantages.

**Table 1:** Summarization of smartphone-based imaging devices for various applications

Applications of smartphone-based imaging devices	Uses	Advantages
Routine diagnosis	Hematological and histopathological examinations Rapid examination of metabolites in blood and body fluids Rapid monitoring for vital health parameters	Portable devices with sensitivity compared to traditional methods Rapid detection, portable devices which are POC in nature Cost-effective methods
Bacterial detection	Fluorescence and autofluorescence capability to monitor bacterial loads Immunoassays for real-time detection of bacterial species Incorporation of analytical platform such as microfluidic devices for high-throughput analysis	Reduces time required for bacterial culture and traditional analytical tests Real-time detection with high sensitivity and specificity
Virus detection	Colorimetric and fluorescence immunoassay for detection of viral strains LAMP and RT-LAMP assays for accurate detection of viruses Imaging nucleic acid molecules to understand the pathology of viruses	Easily quantifiable output directly on the smartphone Less time-consuming than traditional benchtop methods Enabling on-time detection of virus in remote location
Food quality control	Digital image-based cytometric methods for detection of food contaminants Spectroscopic methods for detection of bacterial and viral contamination in food Colorimetric assays for rapid detection of allergens and toxins in food	Faster and rapid detection of food contaminants Pre-screening of live stocks and meat products for quality control Can be used by quality control authorities for regular monitoring
Deep learning for SIDs	Rapid detection of cancers and blood anomalies Studying development in model organisms Useful in non-biological application such as studying air quality, road quality, detecting the efflorescence, and spalling in historical buildings	Increasing capacities of existing SIDs Making the detection and sensitivity of SIDs higher and more accurate Biomedical platforms can be trained as used for non-biological applications Save time, money, and makes the whole analysis process more sensitive

## Disease diagnosis

Routine diagnosis plays an important role in the initial screening of patients at any healthcare unit. High specific detection at a faster rate is desirable, however, with the current benchtop methods, this is attained at a higher diagnosis cost. Also, these are not always present in limited resource areas. With the rapid development of smartphone imaging capabilities, SIDs can be used for various routine diagnosis applications. Microscope-based histopathological examination is considered to be a gold standard for diagnostic examination of various diseases. This requires the preparation of thin stained sections and examination by pathologists to understand the underlying tissue architecture [14]. The process is time-consuming and often examination is not possible in resource-limited settings. Thus, a portable and automated system for the diagnosis would be beneficial. It will save time especially during critical situations like a surgery or an emergency [15]. With the rapid development of smartphone-based imaging capabilities, they have shown to be used for histopathological examination of various diseases. Along with the potential in rapid diagnosis, smartphone-based devices have shown to ease in transmitting the data for further validation [16].

Optical microscopy (OM) since its introduction has been widely used for various diagnostic applications. Its’ role to identify pathological changes in samples with the help of hematological or histological observations has paved an important way in routine diagnosis. Observing the difference among cellular components allows researcher to study and evaluate the integrity at anatomical or morphological scales [17]. Hemocytological examination of blood with an OM-based hemocytometer or using automatic flow cytometry is common for the detection of various diseases and health conditions. The techniques have limitations either in terms of being time-consuming or being very expensive. Recently, cell phone–based microscopy has shown rapid development presenting itself as a ubiquitous platform for cytological studies. Zhu et al. developed a smartphone-based blood analyzer which is portable and uses a very small sample volume of ~ 10 μL for examining the concentration of RBCs, WBCs, and hemoglobin. The device uses three different optomechanical hardware attached to the primary camera of the smartphone to measure the concentrations. The attachment uses 8 LEDs (~ 470 nm) which uniformly excites the fluorescently labeled WBCs, whereas the RBC counter attachment uses a single white LED for bright-field illumination to image unlabeled RBCs. The hemoglobin concentration is determined through absorbance by Beer-Lambert law using a single blue LED (~ 430 nm) to selectively illuminate the cuvette containing lysed blood. The device showed a very high level of sensitivity when compared to commercial hemocytometer [6]. Apart from the above-mentioned use of hemocytological examinations, OM is also used for detecting the presence of various parasites in blood. Filarial nematodes are a major burden to various regions of the world both in terms of socioeconomic and public health. Rapid screening method is often required to detect the level of micro-filarial (mf) in blood in the field. Existing methods include convention pathological examination of stained blood smears using a microscope or using quantitative polymerase chain reaction (qPCR), which is time-consuming and expensive. As an alternative, D’Ambrosio et al. developed a smartphone-based video microscopic device to detect the load of mf in blood based on the wriggling motion of the parasite to confirm its presence. The device referred to as CellScope Loa was built using the reverse camera lens module attached to the rear camera and an LED array to illuminate the sample. The device was compared with standard microscopy and the correlation between both methods was found to be 0.99. The blood smear vs CellScope Loa graph showed the presence of zero false-negative results and two false-positive cases assessed with 99.99% certainty. The CellScope Loa device could also be useful for screening and quantifying trypanosomes, filariae, and other motile blood-borne infectious agents at a rapid rate and high sensitivity [7].

**Fig. 1:**
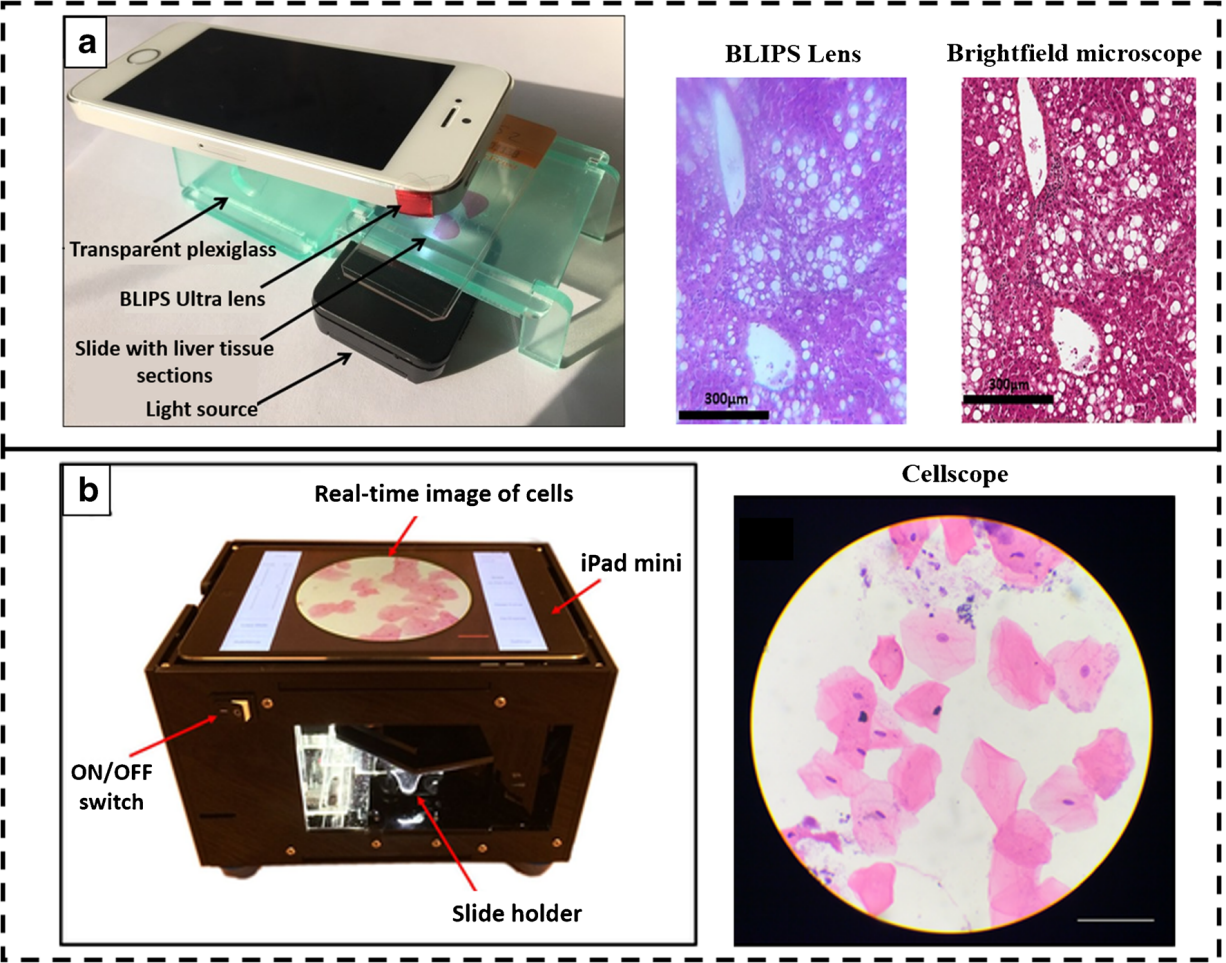
Smartphone-based imaging system. **a** The BLIPS lens-based smartphone microscopic system along with the acquired images of human liver tissue section obtained and comparative image of the bright-field microscope. Reproduced from Cesaretti et al. [20], with permission from Jhon Wiley and Sons (copyright 2016). **b** The real-time image acquisition of cancer cells. Reproduced from Skandarajah et al. [21], with permission from PLOS (copyright 2017)

Histological examination using OM also plays an important part of routine diagnosis. Different microscopic techniques are used for examining the histological slides. This requires extensive microscopy setup as well as an experienced personal for visual examination of the slides, causing hindrance to rapid diagnosis in remote settings [18]. SIDs have demonstrated as point of care (POC) units which can be used for a wide variety of applications along with the ability of algorithms for automatic analysis. Histological examination plays a vital role during organ transplantation to check the liability of the organ. Macro-vesicular steatosis (MS) is often seen as the main reason for liver graft failure due to the accumulation of triglycerides in the cytoplasm of hepatocytes [19]. In a study, Cesaretti et al. developed an add-on BLIPS lens which is attached to the rear camera of the smartphone to perform liver MS assignment. The BLIPS lens was attached to the phone camera with sticky pads and the phone was placed on a stage made of plexiglass as shown in Fig. 1a. The smartphone, white LED as light source, and slides were manually aligned to achieve the best focus at the sample plane. Three-micrometer-thick tissue blocks were stained with H&E stain and used for imaging. MS is histologically characterized by the presence of empty vacuoles in the cytoplasm of the hepatocytes and the percentage of such cells was used to grade the MS. The smartphone system and microscopic approach showed a strong correlation in determining the conditions [20]. The gold standard test for cancer detection is the histopathological examination of suspicious anomalies in the cells. This involves the performance of biopsy followed by examination using OM, which is painful, time-consuming, and requires specialization [22]. The same problem even rises while examining oral cancer patients. Furthermore, due to the presence of a large number of non-specific lesions in the mouth, a more sensitive diagnostic tool could be useful in a rural setting. Skandarajah et al. developed an automated CellScope device for oral cancer screening based on brush biopsy. Figure 1b shows the device which contains a × 20 objective lens that focuses the white LED light on the sample slide and captures the images using an iPad Mini. The samples were collected using Cytobrush Plus GT and cells were released from the brush by titration. The sample was then spread in a glass slide and H&E staining was performed. The application was developed, and 125 random images were acquired from the slide in a raster pattern and only those images which had > 50% cell density were considered. Seventy percent sensitivity was observed in CellScope-based examination as compared to standard histological analysis. Also, the developed device is portable and can prove itself as a POC device [21].

Moreover, routine diagnostics also involves tests which do not require hemato-histological examinations. These diagnostic tests are essential to understand the progression of the health condition or disease state. SIDs have shown to be used for such diagnostic examination which reduces the time and cost of tests. Diabetes is one of the major health-related issues globally. There exist several complications with diabetes and thus require daily monitoring. Soni and Jha developed a SID to monitor the salivary glucose level using a test strip. The strip was made from the Whatman filter paper to which glucose oxidase (GO) enzyme is immobilized. Salivary glucose reacted with GO and causes a change in pH, which was detected due to the color change of bromothymol blue. A smartphone camera was used to detect the intensities of color change using a custom-built application using the slope method. The developed device showed limit of detection being 24.6 mg/dL making it suitable for mass diagnosis in remote areas [23]. Furthermore, for detecting the total bile acids and cholesterol in whole blood samples, a smartphone-based bio-chemiluminescence platform was demonstrated by Roda et al. Bio-luminescence assay using 3α-hydroxysteroid dehydrogenase was performed to check the total bile acids while a luminol−H_2_O_2_−horseradish peroxidase chemiluminescence assay system coupled with cholesterol esterase/cholesterol oxidase was integrated for the total cholesterol measurement. The reactions took place in a mini cartridge containing a blood separator pad connected to the nitrocellulose disk containing either enzymes. This cartridge was placed in a 3D-printed black box and attached to the rear camera of the smartphone and showed adequate analytical performance with good accuracy and precision [24]. While utilizing the capability of smartphone to make it portable and easy to operate for carrying out various bioanalytical assay with very high sensitivity, the smartphone devices have also shown to increase sensitivity to detect even the slightest variation in the results which are often invincible with visual examination.

With the rapid development of smartphone hardware, it is observed that a lot of health monitoring systems are being merged within the smartphone. The recent trend of smartwatches have seen this merger, too, which enables monitoring of heart rate to the latest of being able to perform electrocardiogram (ECG) within the device. However, these high-end smartphones and smartwatches are costly and often not available to the masses. Thus, being able to incorporate health monitoring with mid-range smartphone is the need of the hour. Photo-plethysmography based on a smartphone has been used for measuring oxygen saturation and cardiac rhythm. Chan et al. used it for the screening of atrial fibrillation. The smartphone flashlight is used to illuminate the finger and the reflected light was captured by the rear camera. The signal acquisition time was around 1 min using the developed application. The device showed a diagnostic sensitivity of 92.9% with the specificity being 97.7% and POC in nature [25]. Since no additional hardware is used for monitoring health parameters, smartphone applications are used to derive information which is user-friendly. Other smartphone-based platform is Seismo that uses the accelerometer of the smartphone to record the flow of blood and vibrations from the heart valve movement and measures pulse using the smartphone’s camera in conjugation to monitor blood pressure. The device was compared to standard methods such as ECG or traditional PTT (pulse transit time) instruments and was found to have a high correlation to the previous methods [26]. Both these methods did not incorporate any additional hardware and can be implemented with any smartphone, thereby making it inexpensive and POC devices.

In conclusion, SIDs have been used for various routine diagnosis applications, which otherwise requires sophisticated instruments and are generally time-consuming. These devices help in bringing affordable diagnosis to the masses in remote areas with higher specificity and sensitivity to help in continuous monitoring of the health state with ease.

## Bacteria detection

Detection and identification of bacterial pathogens from biological samples is key to diagnose a myriad of infectious diseases. However, conventional methods including culture-based methods or PCR-related detections are cumbersome, time-consuming, and require adequate resources such as expensive instruments and skilled technicians [27]. SIDs have grown rapidly for various bioanalytical applications and used as POC detection units. Bacterial detection for various clinical and research purposes often includes various analytical tools. Optical imaging methods have proven to be essential tools for screening and detecting a wide variety of bacteria. However, the traditional microscopic devices are bulky, expensive, and require advance laboratory settings for operation [28]. With the increasing demand for POC devices, a paradigm shift is being observed towards the development of cost-effective and portable devices. SIDs have shown capability comparable to that of traditional optical microscopes [29]. Bacteria is in microscale and require high magnification for imaging. Smartphone-based devices show a limitation to attain such high magnifications. Combining with counter-staining techniques such as fluorescence, bacteria can be easily detected and quantified with smartphone devices. Mullër et al. developed a compact smartphone-based fluorescence microscope for imaging fluorescently labeled bacteria. A 488-nm diode laser was used for illuminating the sample at an angle of 61°. The fluorescent signals from the samples were collected using an external lens and passed through a long pass filter before it reached the smartphone rear camera sensor. All the optical components were placed inside a 3D-printed optomechanical box. Bacteria were fluorescently labeled with peptide nucleic acid (PNA) probes for rapid identification and quantification. The device was able to detect bacteria to 10^4^ CFU/mL comparable to conventional microscope as well as able to acquire images of pathogenic *Cronobacter* spp. showcasing its ability to be used in resource-limited settings [30].

**Fig. 2:**
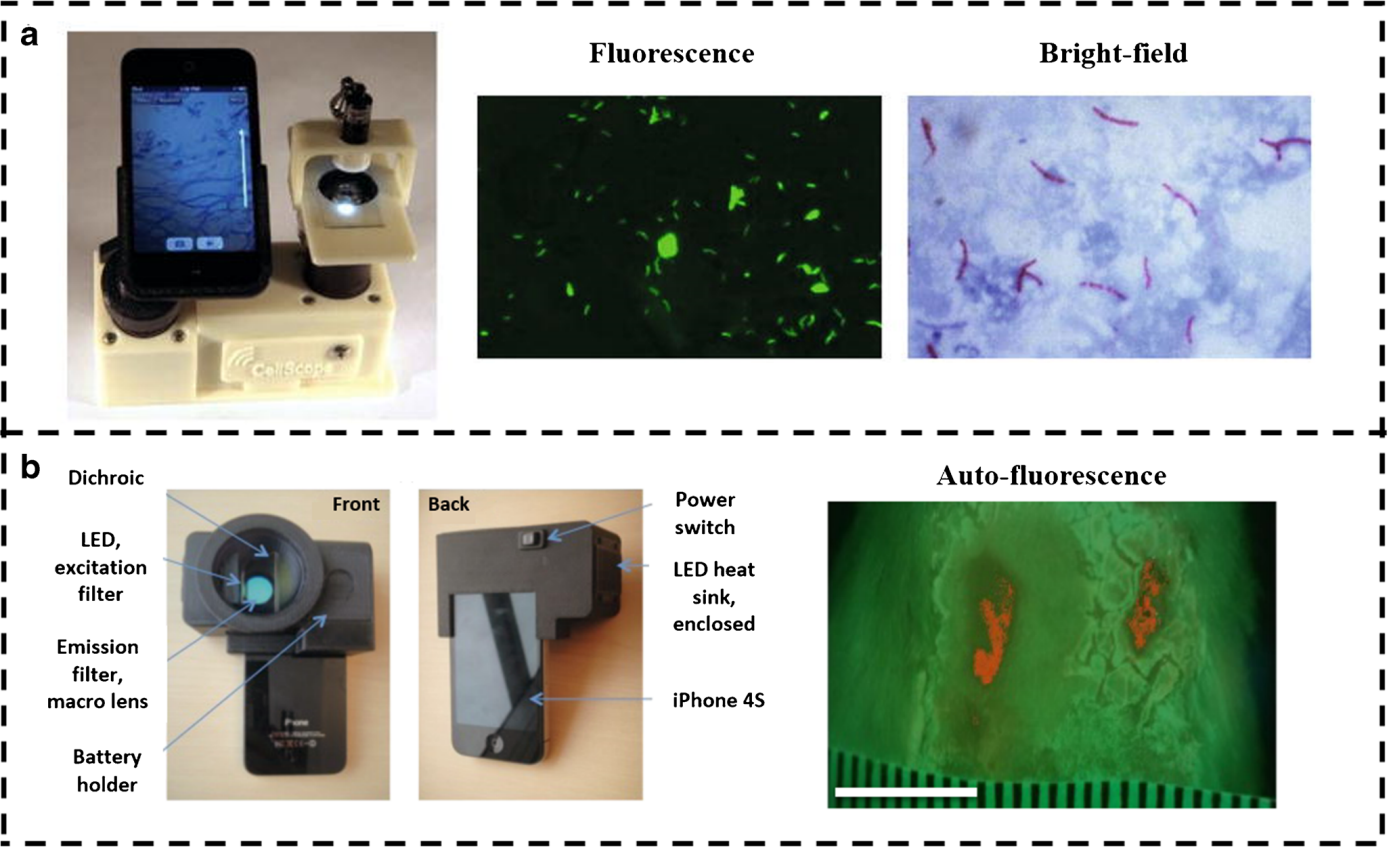
**a** The CellScope system with 3D-printed parts for fluorescence imaging. The fluorescence (green) and bright-field (red) images of TB pathogen, respectively. Reproduced from Chang et al. [33], with permission from Springer Nature (copyright 2012). **b** The PRODIGI system and its components for real-time autofluorescence imaging of wound, collagen (green), and bacteria (red). Reproduced from Wu et al. [34], with permission from Society of Photo-Optical Instrumentation Engineers (SPIE) (copyright 2014)

Optical microscopy is considered the standard technique to examine sputum samples for tuberculosis (TB) and other major procedures involve using PCR and culture assays. Culture assays are challenging to perform and require a few weeks of incubation, whereas the PCR-based method requires expensive setups, thereby limiting its use to high-end resource settings [31]. Therefore, sputum smear microscopy is most commonly used to diagnose TB. Thus, improving microscope-based screening methods could help considerably in diagnosing TB. Fluorescence microscopy has shown higher sensitivity as compared to the bright-field microscope. With the development of the light sources for fluorescence microscopy, the latter is becoming cost-effective and usage is also being increased [32]. Chang et al. developed CellScope, a smartphone-based fluorescence microscopic system for TB detection. Figure 2a shows the device which is made of 3D-printed parts along with an inkjet-printed lens that was attached to the camera of the phone. Multiple LEDs with fluorescent filters were used to excite the samples. As the sample stage is not movable, it prevented the focal adjustment of the sample with the lens. TB objects are classified from the negative ones by support vector machines (SVM) and could attain an average precision of 89.2% ± 2.1% enabling an easier and accurate diagnosis of TB from sputum smear samples [33].

Healing of wounds is usually hindered by the presence of bacteria and it is not always possible to detect these bacteria due to the difficulty in identification and also many patients may not manifest symptoms [35]. Current methods to monitor infected wounds include visual assessment of the wound as well as look for clinical signs and symptoms which are less-effective and time-consuming. Wu et al. developed Portable Real-time Optical Detection, Identification, and Guidance for Intervention (PRODIGI), a handheld autofluorescence imaging device that detects and quantifies the bacteria in wounds in real-time as shown in Fig. 2b. The device being handheld and portable can be easily taken to field or used in house setting for easy monitoring of wound healing. PRODIGI captures both white light and autofluorescence signals from tissue components and bacteria. Broadband white LEDs provided illumination for bright-field imaging, whereas two monochromatic violet-blue LED arrays are used as excitation light sources for autofluorescence imaging. The reflected excitation light from the skin surface is blocked with a dual-band fluorescence filter placed in front of the camera and the autofluorescence signals from bacteria and tissue are separated by an emission filter to display the output as a composite RGB image. The device has shown to be useful in the tracking of wound healing and associated infections, in non-invasive and faster way than other methods [34].

The microscope-based examination shows fluctuating sensitivity rates and requires an experienced personnel for examinations. On the other hand, analytical assay has showed better sensitivity and accuracy in quantitative and qualitative assessment of bacteria. Barnes et al. developed a SIDs for diagnosing bacteria in patients with urinary sepsis. The lysed biological specimen was mixed with a standardized loop-mediated isothermal amplification (LAMP) reaction mixture for the generation of fluorescent signal which was correlated to amplification. The performance of the device was considerable against several gram-positive as well as gram-negative bacterial species comparable to standard assay using a real-time quantitative PCR. The limit of detection was within the range required for positive demonstration of urinary tract infection from clinical culture/samples [36]. The amalgamation of smartphone with LAMP assay increases the sensitivity to detect the outputs as a slight change in read out can be easily quantified with smartphone application along with the ability to read multiplex results at the same time. However, LAMP assay sometimes shows decrease sensitivity with outputs being false-positive. Therefore, nucleic acid–based immunoassay is preferred for rapid detections. Li et al. developed an enzyme-linked aptamer assay complemented with smartphone-based detection for rapid quantification and identification of the various strains of mycobacterium. The bacterium was bound to the nitrocellulose membrane to which biotin-labeled aptamers are attached, by detecting the presence of mannose-capped lipoarabinomannan on the surface of the bacteria. Then, streptavidin-labeled horse radish peroxidase conjugates with the aptamer and develops color in the presence of H_2_O_2_. The smartphone was then used to detect and quantify the bacteria and the technique showed a detection limit of 10^4^ CFU/mL of samples. This showed a higher sensitivity and specificity compared to traditional acid-fast staining assay [37].

Apart from the conventional technologies for analytical purposes, microfluidic devices have appeared to be useful for various analytical and bioanalytical-based detection applications. Microfluidic devices have miniatured many of the bioassay instruments into a single platform as well as reduced the time and increased the throughput of assays. Furthermore, the amalgamation of microfluidics technology with a smartphone has led to the development of a POC diagnosis system. Microfluidic platform can be made from a number of materials but paper-based microfluidic devices particularly have shown to be portable, low-cost, and used for various biochemical assays [38]. Park et al. developed a smartphone-based microfluidics system using cellulose chromatography paper for the detection of various pathogens. Agglutination reactions were performed using anti-*Salmonella*-conjugated polystyrene beads and Mie scattering was used to detect it. The intensity of scattering is directly proportional to the bacterial load and the detection limit with the instrument was found to be 10^1^ CFU/mL (95% confidence interval) with the detection time being less than a minute [39]. Dönmez et al. demonstrated a smartphone-based dark-field imaging device to measure bacteria and bacteriophage interaction. Overcoming the traditional methods to check this interaction which was laborious and took 24–48 h, the smartphone-based device provided results within 4 h. A melt-extruded fluoropolymer-made microfluidic device containing 10 microchannels used to detect the lysis activity was placed into 3D-printed imaging box containing LED inclined at 15° providing illumination. The device was able to detect O.D_600_ of 0.1 corresponding to 8 × 10^4^ CFU/microdevice with a high sensitivity compared to the traditional spectrometer [40]. Microfluidic methods accompanied with smartphone detection system have shown the ability to increase throughput of the microfluidic device by enabling multiplexing and increasing the speed of detection. These devices have highlighted some of the vivid applications of SIDs in the field of bacteria detection, which is very important for monitoring disease progression. SIDs can detect the presence of bacteria much faster and with a lower detection limit at a much lesser cost and are mostly used in complementation with various techniques to provide read out for the experiments. However, differentiating between species of bacteria often comes as a disadvantage with such devices and requires the incorporation of several conventional methods such as antibody-based detection.

## Virus detection

**Fig. 3:**
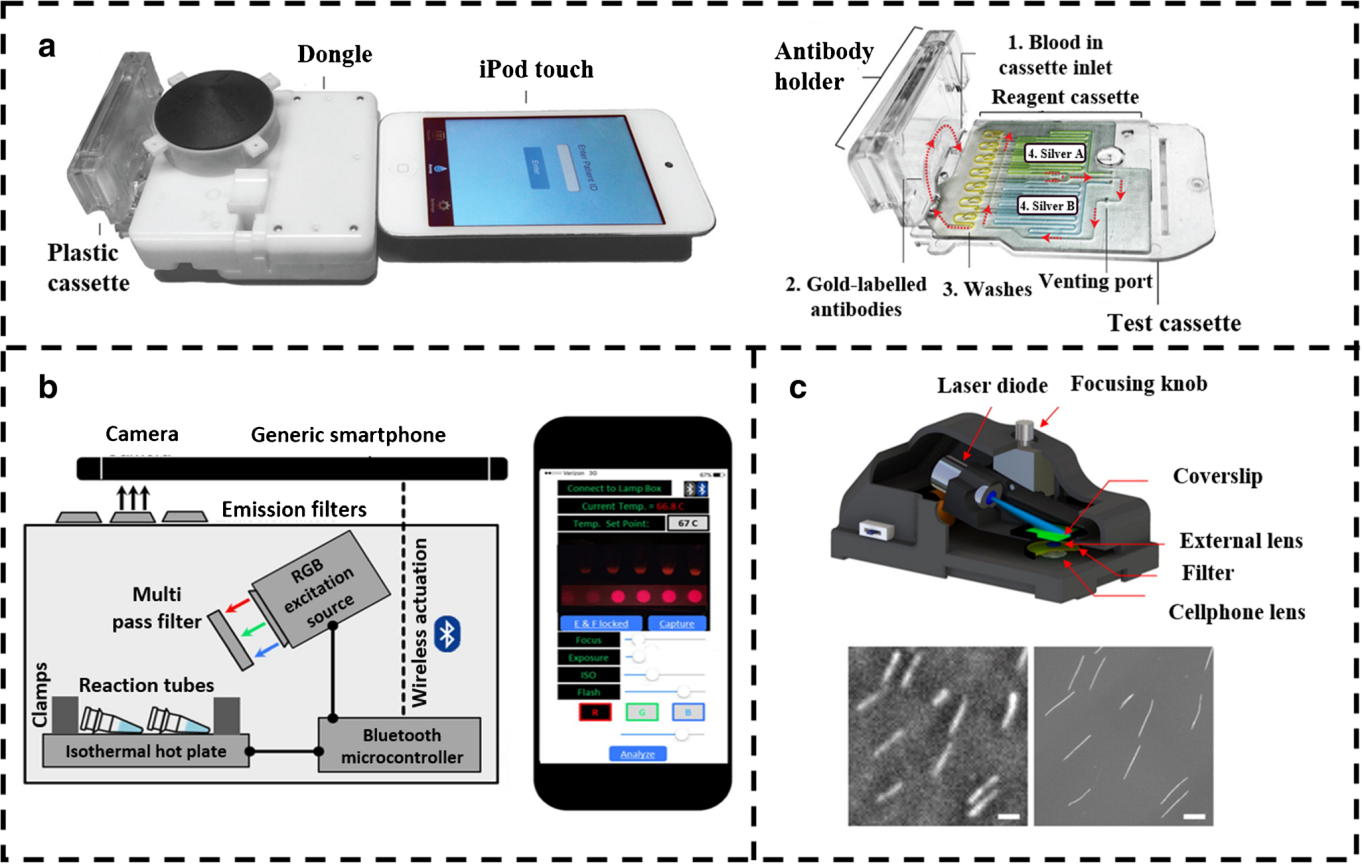
**a** The smartphone-based immunoassay platform for the virus detection integrated with the microfluidic cassette containing the preloaded enzymes and antibodies. Reproduced from Laksanasopin et al. [43], with permission from American Association for the Advancement of Science (copyright 2015). **b** The schematic of the smartphone-based RT-LAMP device along with the smartphone application for real-time monitoring of the reaction. Reproduced from Priye et al. [44], with permission from Springer Nature (copyright 2012). **c** The schematic representation of smartphone-based fluorescence platform for DNA imaging and sizing. The image shows the fluorescence image of the DNA acquired with the device. Reproduced from Wei et al. [45], with permission from Springer Nature (copyright 2017)

Viruses are sub-microscopic, pathogenic particles with either DNA or RNA as genetic material. Most of them use the host cell machinery to replicate and in this process kills the cell. Viruses are known to cause several high morbidity diseases related to both animals and plants and often detection requires specialized tools and processes which are normally not available at primary screening centers [41]. Viruses are mostly screened using various analytical methods which includes immune assay, biochemical assay, or PCR-based methods. Even though these methods have higher sensitivity, their use in remote location often hindrance due to lack of proper resources. Thus, researchers have begun to look towards the development of POC devices with similar efficiency and sensitivity. SIDs are thereby helping to bring the sophisticated laboratory technologies to the field, which will help diagnosis to reach to masses [42]. Laksanasopin et al. developed a dongle attached to a smartphone that mimicked all the actions of a conventional benchtop ELISA instrument, making it another POC diagnostic device being easily available. It was designed to perform a triplexed immunoassay for the detection of three infectious disease markers (HIV, treponemal syphilis, and non-treponemal syphilis). The incorporation of microfluidics capability with smartphones aided in making it cheap and accessible especially in resource-limited settings (as shown in Fig. 3a). The device is portable and uses disposable plastic cassettes preloaded with the required reagent to perform immunoassays using gold nanoparticles and silver ions as the substrate for ELISA. The dongle consumed less power and the use of additional battery was avoided by using the audio jack in the smartphone for transmitting power to it. The results were found to be comparable to standard detection tests and sensitivity of 92–100% was observed, showing immense advantages in terms of disease detection and making them accessible to most of the population [43]. However, this requires complex device fabrication and extensive reagent arrangement in the cassette. Furthermore, to make the immunoassay more simpler, lateral flow devices are used which have enzymes embedded in the membrane. Yeo et al. developed a smartphone-based fluorescent lateral immunoassay to detect avian influenza (AI) virus strains. AI viruses are highly pathogenic and have shown to cause across species infections including in human, and timely detection and controlling of the virus is important. A smartphone-based fluorescence device has already shown its capability in the detection of various viruses. The smartphone detection system consists of a 3D-printed module and LED (excitation wavelength ~470 nm). The strip for the immunoassay was prepared from nitrocellulose and coated with an anti-influenza antibody. Furthermore, bioconjugation of the strip was done using dendrimer before introducing the sample. The smartphone-based device was able to detect H5N1 type with 96.55% sensitivity and 98.55% specificity. The use of smartphone detection method takes around 15 min to analyze the results, which was faster and rapid than most of the benchtop methods [46].

Immunoassays demonstrated higher sensitivity with better accuracy to provide faster results than conventional biochemical tests but often lack specificity. Thus, PCR-based detection methods are observed to provide better outcome. Also, PCR-based method is a time-consuming, complex, and expensive process, thereby rendering sub-optimal use [47]. Therefore, reverse-transcription loop-mediated isothermal amplification (RT-LAMP) has emerged as an important method for such detection in resource-limited settings due to its portability, sensitivity, accuracy, and most of all its capabilities despite such simplicity. Ganguli et al. have developed a multiplexed smartphone-based microfluidics and RT-LAMP detection technique for the Zika virus along with Chikungunya and Dengue viruses. The developed module consists of a microfluidic chip made up of polydimethylsiloxane (PDMS) and consists of three inlets for—blood sample, lysis buffer, and amplification reagent. The blood sample is lysed and mixed with amplification reagents and is then transferred to the chip (that contain RT-LAMP primers for different pathogens and were pre-loaded). Once the smartphone and microfluidics card were in position, fluorescence imaging was initiated which collected the fluorescence signal from the primers specific to the pathogen in blood. The detection limit was found to be 1.56 × 10^4^ PFU/μL for Zika virus in the whole blood sample and 1.56 × 10^4^ copies/μL of purified RNA for Chikungunya and Dengue from the whole blood sample [48]. However, RT-LAMP method usually involves amplification using non-specific indicators of total DNA synthesis; it is necessary to open the reaction tube for post-reaction analysis and hence introduces the risk of amplicon contamination and other complications as well [49]. To overcome the problems stated, Priye et al. developed a method that was target-specific and a multiplexable format of smartphone-integrated RT-LAMP for Dengue, Chikungunya, and Zika involving closed-tube-based experiment. The detection involved the use of “quenching of unincorporated amplification signal reporters” (QUASR). QUASR comparatively has brighter signals that can be detected by the normal human eye or even with a smartphone camera as shown in Fig. 3b and has lower detections of false-positive amplification. It also enables multiplexing targets in a single reaction. The device consists of three main components: heating module, assay reaction housing module, and an optical detection module. Using the Zika-specific primers and without any sample preparation, the virus detection rate at 10^3^ PFU/mL was found to be 100% specific [44]. The smartphone-anchored system can be used for qualitative detection, thereby also making it easier for non-experts to use. The outputs are easily analyzed using smartphone application which also enables sharing the results with experts in a different location promoting cross-validation.

Furthermore, imaging DNA molecules is of considerable importance in diagnostics especially in diseases caused by viruses and other pathogens which involving changes in structure and form of DNA [50]. Able to image nucleic acid also helps in understanding the pathology and the life cycle of the viruses. Conventional optical imaging methods involving complex and expensive setups such as confocal fluorescence microscopy and super-resolution microscopy are usually used to study this [51]. Smartphone-based devices have shown advancement in imaging sub-microscopic entities with the addition of various optical attachments. Wei et al. developed a method that overcomes these hurdles and allows smartphones for imaging single molecules. As shown in Fig. 3c, the device uses 3D-printed optomechanical part integrated with the smartphone camera for fluorescence signal detection. A 450-nm laser diode was used as the excitation source and fluorescence signal was recorded by the CMOS sensor of the phone with an external lens. Two 500-nm filters were used between external lenses and smartphone camera lenses to block scattered background light. DNA molecules of various sizes were labeled with an intercalating dye (YOYO-1). DNA sample was warmed and then immediately cooled to open up the sticky ends and was then placed between 2 coverslips which causes linear stretching of the DNA. The length of DNA was measured using a specialized MATLAB program which gave an accuracy of < 1 kb for 10 kb or longer DNA molecules. It is to be taken on board that the smartphone-based devices for virus detection can provide high sensitivity and specificity which tends to be useful during a viral outbreak. With the rapid development of smartphones, these technologies will be more efficient and also help in the centralized monitoring of diseases [45]. SIDs have hereby shown its application in the field of virus detection and testing. They increase the efficiency and speed of diagnosis with a high level of sensitivity. The use of smartphone for detection of virus shows the possibility to use such device in remote location in times of pandemic. The specificity of detection is a drawback for such technologies and a lot of testing and validation of the devices are still required before they are made available to the masses.

## Food technology

**Fig. 4:**
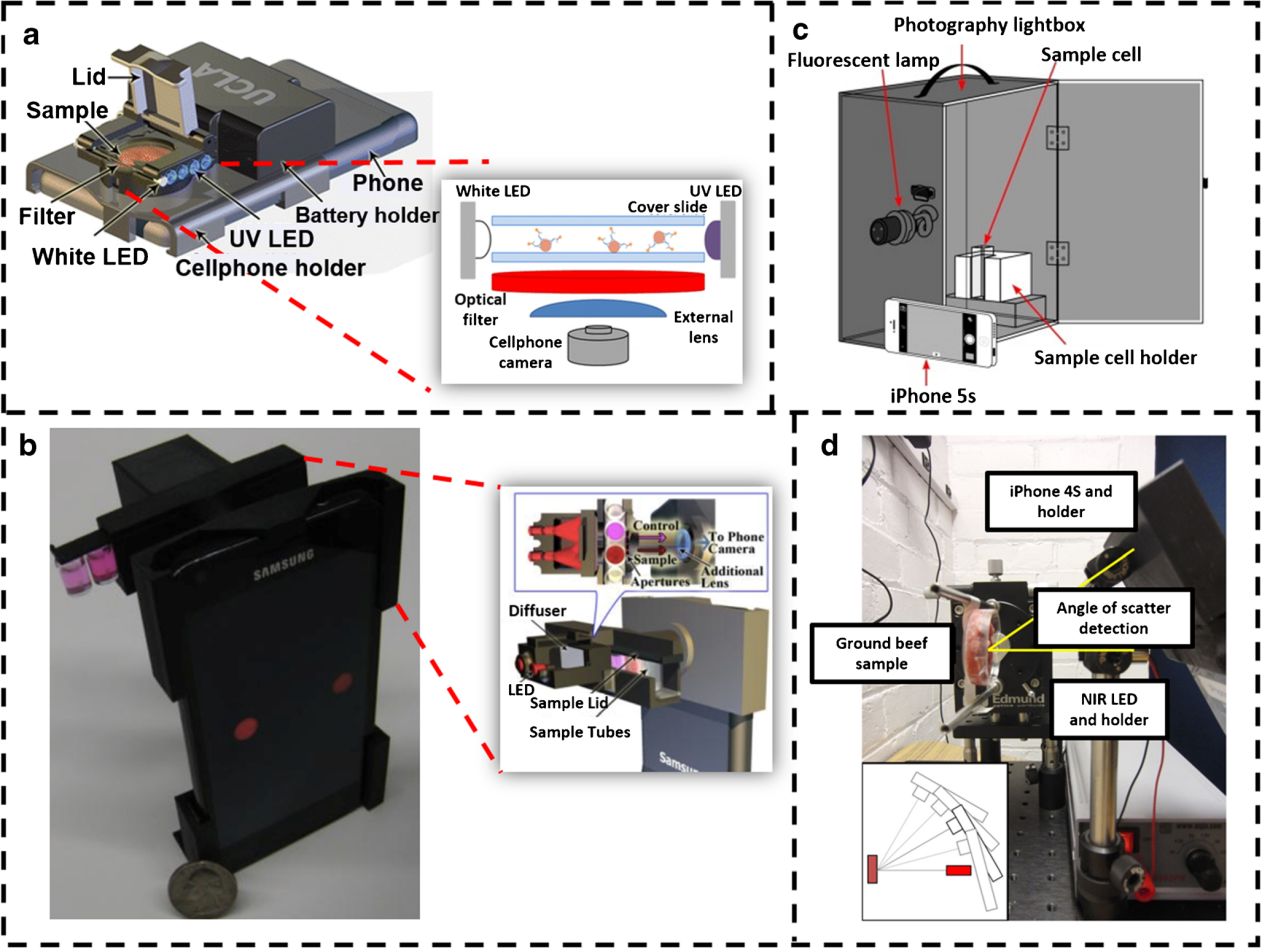
**a** Smartphone-based protein microarray with 3D-printed attachment and respective schematic of the detection platform. Reproduced from Ludwig et al. [54], with permission from Springer Nature (copyright 2014). **b** The schematic representation of the fluorescence colorimetric assay platform based on smartphone. Reproduced from Masawat et al. [55], with permission from Elsevier (copyright 2015). **c** The prototype of NIR smartphone-based tool utilizing Mie scattering for detection of bacteria in beef sample. The small box at the bottom shows the different angles utilized by the device for detection. Reproduced from Liang et al. [56], with permission from Springer Nature (copyright 2014). **d** The smartphone-based iTube platform for performing colorimetric assay. The enlarged region shows the optical arrangement and the detection module of the device. Reproduced from Coskun et al. [57], with permission from Royal Society of Chemistry (copyright 2012)

Food is one of the basic essential needs of every human and various regulations are enforced for maintaing the quality and preventing any health hazard or sickness. Monitoring of contaminants in a food chain is performed by taking the samples and analyzing it in control laboratories; however, it is costly and usually time-consuming. With the rapid development of smartphones to detect bacteria and viruses, they have also shown to be used for various analytical applications in food-monitoring technologies [52]. Food contamination is a major concern to the human kind and ignorance may lead to causality. Milk is one such product which often contaminated with hormones that are given to the animals to increase their output. These have shown many adversaries in humans particularly in children [53]. To detect such hormones rapidly, Ludwig et al. developed an effective and low-cost method for pre-screening recombinant bovine somatotropin (rbST) contaminants in milk using the microsphere-based flow cytometric immunoassay (FCIA), with a cell phone–based platform as shown in Fig. 4a. The 3D-printed attachment cell phone module consisted of 12 LEDs (λ = 380 nm) for fluorescence imaging, 2 white LEDs for dark-field imaging, and an optical filter for blocking the scattered excitation light. This device lead to a new field of imaging in food technology, as it was the first device where both dark-field and fluorescence imaging were integrated. The developed assay was compared to a laboratory-based FCIA method and it was found to predict 95% of the samples correctly. Based on this, it can be deduced that the accuracy was appreciably close to laboratory results, enough for pre-screening showing the efficacy of the device [54]. Such devices catered for everyday use has bridged the gap between what is prepared at laboratories and what can be used for everyday purposes. Besides hormones, it is also been reported that farmers use a lot of antibiotics to protect the livestock from diseases and infections. The antibiotics are used in amounts more than prescribed and traces are found in various animal products. This is observed to be one of the reasons for the rapid antibiotic resistance in the human population [58]. Thus, rapid detection of these antibiotics is needed. Masawat et al. proposed a digital image colorimeter as shown in Fig. 4b, wherein the analyte of interest, tetracycline, was detected for antibiotic residue detection in milk. The image was taken by ColorConc application on the iPhone camera through a drill hole of a black-painted box consisting of the solution of interest inside. The device was able to detect tetracycline in the range of 0.5–10 μg mL^−1^ with the limit of quantification 1.5 μg mL^−1^ with high sensitivity and rapidity [55]. Another application of smartphone-based biosensing was developed for the detection of clenbuterol (CLB). CLB is an illegal additive in livestock feed that persists in animal tissues, and once consumed, the human experiences symptoms of chest pain, increased heart rate, and temporary dizziness. The method specified is different from the other methods as here; detection was done using immunoreaction. The substance was detected from a layer of immunoglobulin using modified multi-walled carbon nanotubes, a screen-printed carbon electrode (SPCE) along with a layer of immunoglobulin. CLB detection ranging from 0.3 to 100 ng mL^−1^ was attainable with the developed device [59].

Food contaminated with bacteria is one of the leading causes of food poisoning and often show severe symptoms like diarrhea and vomiting. Contaminated food can reach the population from the food vendors and restaurants. Thus, it is essential to check for the presence of pathogens [60]. Most of the developed methods to detect pathogens in food involve culturing them and then detection using various standardized tests, which is time-consuming. Smartphone-based devices have shown to detect various contaminations. Liang et al. applied a smartphone-based spectroscopic method for detection of microbial contamination in beef samples based on Mie scattering, in which the scatter signal intensities were detected at different angles (15°, 30°, 40°, 60°) as shown in Fig. 4c after the surface of the beef sample was irradiated with 880-nm near-infrared LED. *E. coli* concentrations in the range 10^1^–10^8^ CFU/mL were successfully quantified. The non-usage of antibodies in this experiment showed that similar bacterial species could not be distinguished [56]. Bueno et al. used smartphone technology to check the presence of foodborne pathogens in meat by detecting the presence of three profiled amines (triethylamine, isobutyl amine, isopentylamine). Cellulose membrane was immobilized with five different pH indicators and all of them were exposed to each of the amines to generate color profiles. The smartphone was used to capture the images and quantified using principal component analysis (PCA) and hierarchical cluster analysis (HCA) analysis. The method was able to quantify amines up to 1 ppm showcasing the ability to provide early detection of pathogens [61]. While it is possible that these pathogens do not harm certain people, detection of these pathogens is vital as they can prove it be harmful to others.

Food allergy is another major problem to be tackled in order to maintain a healthy lifestyle. Often, various foods or food contents give rise to allergy in different people. Also, it is one of the growing causes of modern health problems worldwide. Certain fishes, nuts, lactose, glycan, certain chemicals, etc., are considered as common allergens. The content of such allergens is necessary to be mentioned in any food products but these data are often manipulated for business purposes. Thus, detecting such allergen faster than traditional laboratory-based methods is often admired. In a study, Mora et al. designed a smartphone attachment that could measure lactose or galactose concentrations in food samples. They used *E. coli* as a marker that was genetically modified to fluorescence when the target molecules were present as substrate. Smartphone cameras were used as detectors for colorimetric-based measurement in determining the concentration of specimens in the range of 1–1000 nM, a high sensitivity due to the use of fluorescence imaging. This living material–based analytical sensor (LiMBAS) used biomaterial that led to establishing the growth of microorganisms outside laboratory environment [62]. Coskun et al. assembled a colorimetric device iTube that could be coupled with a smartphone to detect traces of peanut in food samples. Packaged food labelling will not necessarily include traces of food samples but might contain allergens that could prove harmful to people that consume it. While there are fewer alternatives to such portable devices, such a device could prove to be pioneering. Two test tubes (food sample and control) were illuminated with the absorbance wavelength of 450 nm, and the light absorbance was computed using an application in the cell phone as shown in Fig. 4d. The detection limit was found to be ~ 1 ppm and the time required to conduct the test (from sample preparation to detection) was around 20 min paving the way for faster detection of food allergens [57]. A similar principle was followed to quantify aflatoxin in maize, a toxic secondary metabolite [63].

Drug abuse is another major issue that needs to be dealt in a global level. In contrast to detection of contamination in milk where only one biomarker is used, for detection of drug abuse in sports doping and veterinary control, a single biomarker is often not considered as ample. The author has taken into consideration of various confounders like age, weight, and gender and introduced the idea of multiple biomarker detection to eliminate such factors. Ludwig et al., hence, proposed an attachment-based system that takes less acquisition time, sample volume, and is cost-effective for the simultaneous analysis of biomarkers. Drug abuse detection requires an additional step of biomarker identification. This is what separates detection in athletes and animals. The detection of rbST was performed with two rbST-dependent biomarkers—rbST antibodies and insulin growth factor1 (IGF-1) antibodies. The same smartphone fluorescence microscope attachment as mentioned previously for the detection of rbST was used along with a 48-pore microarray. The difference in intensities of untreated and treated samples is obtained from the microarray after imaging to quantify the levels of drug [64].

Overall, the role of smartphone-based imaging devices can be recognized as a potent tool in the field of food technologies to deal with the complications which can be life-threatening. They also provide efficient and rapid monitoring as an extensive measuring tools which makes the method portable as well as cost-effective.

## Deep learning for smartphone-based imaging device

Optical microscopic imaging techniques are widely used in bio-optics and biomedical fields for the analysis of biological samples. Although it is a gold standard technique, it has certain limitations such as instrumentation cost, portability, the requirement of expertise in the field for analysis of sample, and maintenance. On the other hand, smartphone-based imaging is an emerging filed, which can be an alternative solution for most of the limitations of laboratory optical microscopes. However, poor image resolution is a major drawback of smartphone-based optical imaging which can be resolved using an efficient image processing technique such as deep learning (DL) [65, 66], which uses multiple layers of neural networks for the data abstraction. These techniques are automated and provide accurate outputs with minimum human intervention. Hence, smartphone-based microscope systems employed with deep learning techniques are used in various fields from cancer to environmental research [67–73].

**Fig. 5:**
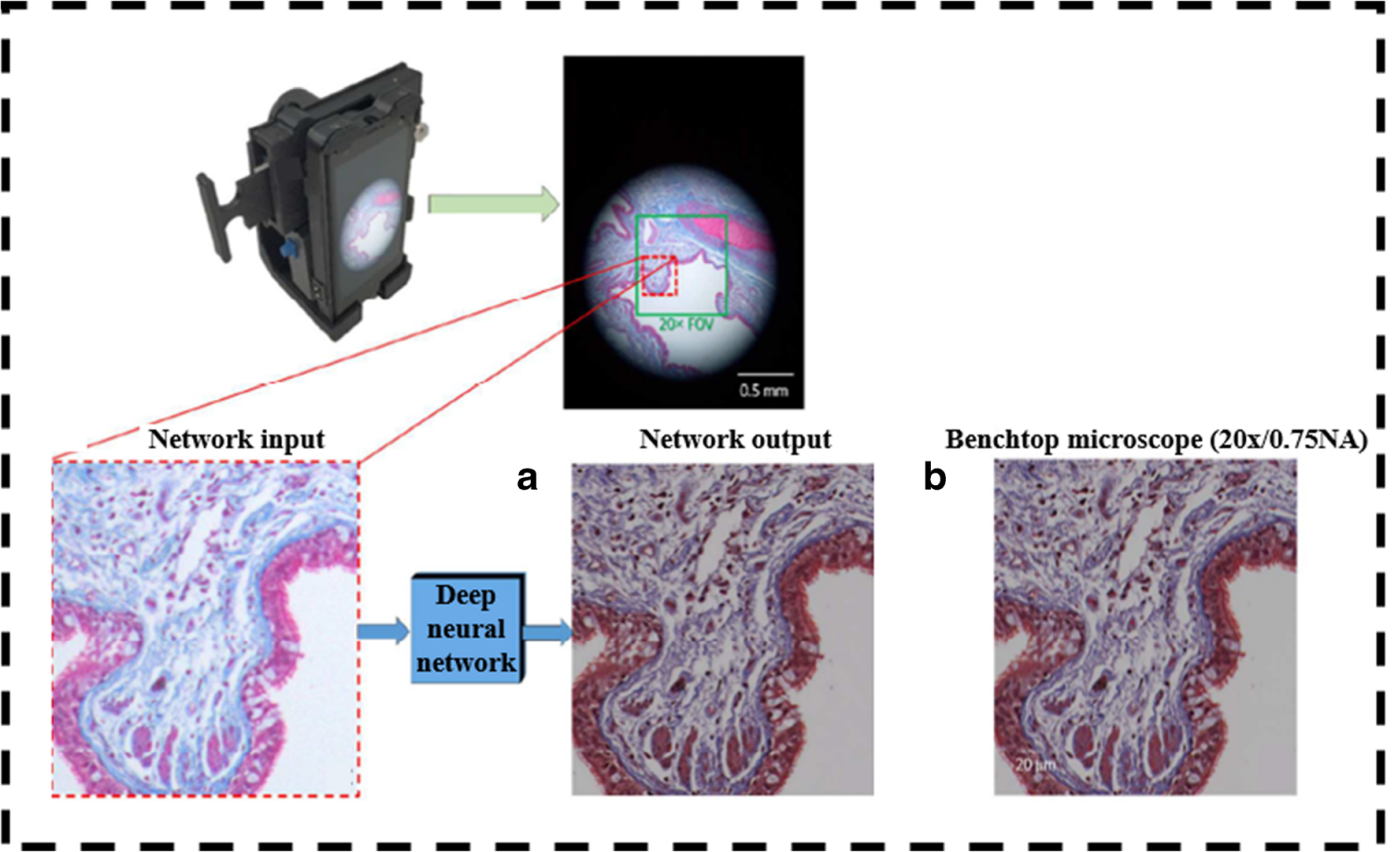
The smartphone-based microscopic imaging system complimented with image processing utilizing deep leaning–based CNN. The images marked as **a** and **b** shows the comparison of deep learning–enhanced image to that of benchtop microscopic image. Reproduced from Rivenson et al. [65], with permission from American Chemical Society (copyright 2018)

Among various DL algorithms, convolutional neural network (CNN) is used as a potential tool to enhance the image resolution. Rivenson et al. developed an experimental setup consisting of a Nokia Lumia 1020 with a CMOS image sensor and an additional five plastic lenses along with built-in six lenses of a smartphone’s rear camera as shown in Fig. 5. A 12 RGB LED ring structure is used to illuminate the sample and a 3D-printed optomechanical attachment with XYZ stage adjustment for a sample. The different characteristics from the input RGB image are extracted using the convolutional layer of the deep CNN and rectified the linear unit as an activation function. Initially, multiple networks with the same architecture are trained using lung tissue, blood smear, and Papanicolaou samples. During the training phase of the algorithm, images from a smartphone-based microscope are compared with × 20 resolution images of Olympus IX83 microscope and then the cost function is calculated in each iteration to update the network parameters using a backpropagation algorithm. Later, these networks are blindly used to analyze different pathological samples. Further deep learning techniques also corrected color distortions caused by incoherent light sources used in the experimental setup [65]. The same group of researchers developed a DL model to identify the sickle cells from the peripheral blood smear. The model enhanced the smartphone images and later segmented the sickle cells from the same model. The overall accuracy of the model was observed to be 98% and, hence, was proved as a potential tool in screening the disease in resource-limited regions around the world [67].

**Fig. 6:**
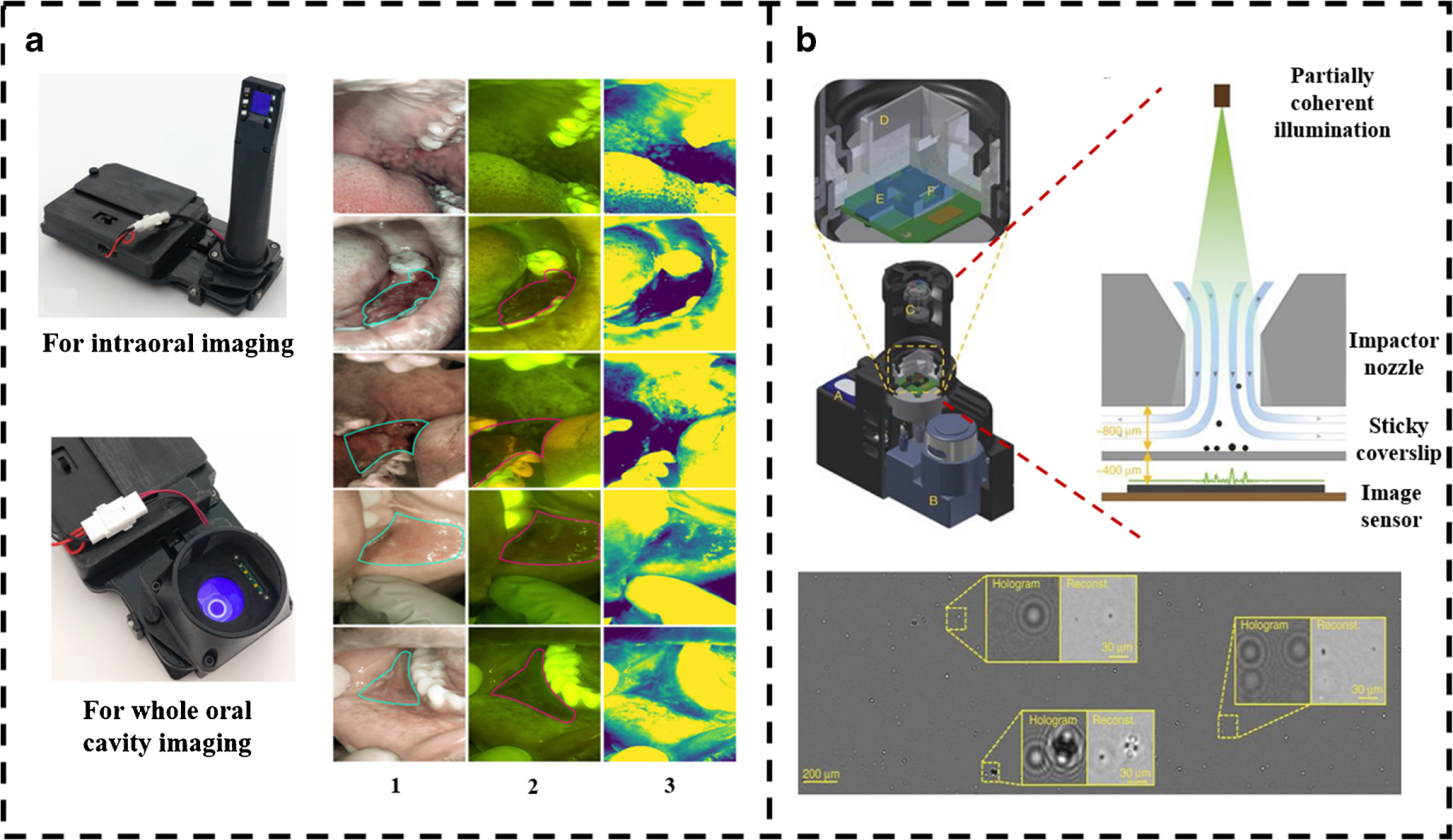
**a** Prototype of smartphone-based imaging platform showing the attachments for intraoral imaging and whole cavity imaging. The first column of images is white light–based, whereas the second column is autofluorescence-based. The third column shows the green intensity map of the respective images with the mean subtracted. Reproduced from Utoff et al. [68], with permission from PLOS (copyright 2018). **b** Identification and quantification of particulate matter with the help of the smartphone base c-Air device. Reproduced from Wu et al. [73], with permission from Springer Nature (copyright 2017)

Cancer is one of the life-threatening diseases, demanding a potential tool for early diagnosis. Smartphone-based microscopy can be used in the assessment of cancer, when employed with DL technique for image enhancement and classification. In this direction, researchers developed a smartphone-based microscope with the android application to identify oral cancer in remote areas. The device consists of an LG Android smartphone with an intraoral and whole cavity imaging facility as shown in Fig. 6a. Both bright-field and autofluorescence imaging are performed using this device, where auto-fluorescent imaging helps in the early detection of oral cancer by imaging biomolecules present in it. The images are uploaded to cloud servers using Wi-Fi and later they are classified using a pre-trained CNN as well as diagnosed by a remote specialist. The summary report consists of data uploaded from a smartphone, CNN result and diagnosis from a specialist, and is viewed and downloaded at any point in time from the cloud server. The sensitivity and specificity of classification in the case of remote specialists are 0.9259 and 0.8667, respectively, whereas for CNN, it is found to be 0.8500 and 0.8875 [68]. Also, the development is made in the direction of assessing the quality of life of cancer patients with the help of a human dynamic reporting service (HDRS) application, which monitors the activities of the patients based on the data acquired by their mobile phone sensors [69]. Furthermore, in a study conducted by Alzubaidi et al. (2019), a CNN model DFU-QNET was used to grade the severity of foot ulcers in diabetic patients into two classes known as normal and abnormal. Several other pertrained networks are also considered and trained with transfer learning technique and their performance is compared with the novel model. The smartphone-based imaging techniques are also employed in detecting drug abuse with the automatic machine learning detection method [70].

Apart from biomedical applications, smartphone-based microscopic setup employed with the support vector machine (SVM) algorithm is used in automatic detection of the *Caenorhabditis elegans* (*C. elegans*). Initially, *C. elegans* wild-type worms were cultured and are imaged using a Samsung S7 smartphone equipped with an additional lens of 12-fold magnification. A set of 12 light-emitting diodes (LED) arranged in 4 by 3 array are used as a light source for a smartphone-based microscope. To train the SVM algorithm, a total of 240 images of Petri dishes are taken and features from the acquired image were extracted using histogram of orientation gradient (HOG) for image pre-processing. The extracted features are used to train the SVM algorithm for the detection of *C. elegans* in the petri dish. The whole process is automated using the smartphone application which provides the user interface to capture, store, and process the images. This technique showed 90% sensitivity and 85% specificity with pre-processed data. The miss classification rate was 12.5% due to false-positive values caused by scratches, adhesive tapes, or inscriptions on the plate that can be solved by taking certain precautions and an increase in the window overlap increases sensitivity [68]. In another study, an AI-based mobile application platform called a mobile water kit (MWK) is used to identify bacterial contamination in water. Android Studio, an open platform, is used to build the mobile phone application, through which the sample images are captured and later classified using cloud-based CNN and the response was sent back to mobile for further analysis [72].

Smartphone-based imaging is also found effective in assessing the air quality by measuring particulate matter (PM) in the air. The study is conducted around Los Angeles International Airport (LAX) by monitoring the quantity of PM in air using a smartphone-based lens-free microscope with machine learning technique. This device is called c-Air which consists of an impaction-based air sampler to capture aerosols. A laminar airstream is driven by the pump at high speed through the nozzle against a sticky coverslip which helps to collect the aerosols and used for imaging as shown in Fig. 6b. Machine learning algorithms are used in particle detection and sizing. The device is capable of generating microscopic images and PM sizing by screening 6.5 L of air in 30 s. PM detection is very crucial since it can impact a severe health problem. Further c-Air PM measurements are closely correlated with US Environmental Protection Agency (EPA)-approved standard techniques such as beta-attenuation monitoring (BAM) and tapered element oscillating microbalance (TEOM) [73]. The neural network–based systems are also employed in automatic detection of road cracks and estimation of the amount of dust on a gravel road which assures the driving safety on the road [74]. Furthermore, a faster R-CNN model is used to detect the efflorescence and spalling in historical buildings to overcome the manual examination error which may destroy masonry structures [75]. They are also employed in detecting drug abuse with the automatic machine learning detection method [76]. Thus, a smartphone-based imaging being a cost-effective, portable, and powerful technique can have many more useful applications in the coming days. It has shown promising results when employed with deep learning image processing techniques for analysis, particularly in remote areas and also during medical emergencies; furthermore, when the complete medical assistance is not easily available, these smartphone-based microscopes can be used to analyze the disease conditions in a short interval of time, hence reducing causality [77].

## Conclusion

 SIDs have shown to be a powerful tool for biomedical research. It had undergone continuous development with the integration of different types of microscopic and spectroscopic techniques to gain rapid appreciation in the market. Most of the devices use smartphone camera as the detection system which gives the user ubiquitous ability for colorimetric, fluorescence, absorbance, luminescence, and reflectance measurements. SIDs have enabled research in the fields of material science, cell biology, virology, and other related areas to explore and discover the unknown. From the detection of various disease-causing pathogens to food science and education, these devices have shown numerous possibilities [78]. Embedding new sensor and applications with smartphones have given the ability to measure more physical quantity today than it was 10 years before. Further being connected to several wired and wireless connections also gave SIDs the advantage to record data automatically on cloud servers or share them in secure platforms for expert analysis. The simplicity, low-cost, portability of SIDs and availability of user-friendly application make them an attractive alternative with possible prospects being incredible. The added advantage is that analysis is faster and they can be used even without the presence of a trained personnel.

From this article, it can be envisaged that the smartphone-based devices have demonstrated to be highly sensitive and rapid platform. Table 2 shows the comparison of different SIDs for vivid biomedical applications. SIDs have shown its potential for hemato-histological examination or detection of bacteria with high sensitivity and concordance with the standard techniques of OM. Overcoming the conventional techniques for bacterial detection, SIDs are integrated with technologies like LAMP, fluorescence, and autofluorescence to detect and quantify the bacterial load in real-time providing a faster prognosis. These devices have also improved sensitivity of virus and contaminant detection as well as reduced detection time with efficient assays. Various research groups have demonstrated the use of smartphones for the detection of fruit ripening, detection of galactose content, and peanut traces in food items. With the advancement of technologies and ability to miniaturize complex components such as lenses and lasers, SIDs can be used for various biomedical applications as a POC device. With the amalgamation of analytical methods such as Raman or Fourier transform infrared spectroscopy with smartphone, it will have great importance in everyday life for detection of bacteria or contaminants in food and water to detection of harmful and hazardous chemicals. Integration of novel technology such as optical coherence tomography or endoscopy with smartphone platforms will be able to provide faster care through diagnosis using tele-laboratory services even in small health clinics. The ambient light sensor which is basically a photodiode is usually used to regulate the brightness of the display based on the surrounding. It has been used as a spectrometric system similar to the laboratory-based systems to detect contaminants in water and to monitor various colorimetric assays [88]. Further microfluidic devices which are able to carry out various biochemical assays in POC setting, their integration with SIDs has shown to be essential for bringing diagnosis to house settings. Deep learning–based image processing techniques are also implemented that can efficiently make the process of detection and obtaining read-outs more efficient and rapid. SIDs can be used for educational purposes as devices or to carry out various assays in remote schools eliminating the need of expensive instruments. As smartphone connectivity is increasing very rapidly and with their increasing use as remote educational tool, they can be further used for practical learning where the teacher monitors practical being performed from their home in times of global pandemic situation like COVID-19. The future of smartphone-based analytical and bioanalytical devices will depend on the ability to perform diagnostics and health monitoring from a wide variety of samples with similar efficiency and minimal processing. Other direction could be bringing daily health monitoring in the hand of general people. It will help in regular management of health, thereby saving lives from adversaries which often remain unseen for being non-symptomatic or delayed onset. Users can also detect any contaminants in the food while they are at restaurants or be able to determine the various components in the food with smartphone-based analytical imaging devices, thereby reducing changes of allergies. Having such sensitive bioanalytical capabilities in the field could also lead to on-the-spot tracking of contamination and combining the phone’s GPS data with biosensing data to map the spread of pathogens. SIDs are also used for other applications like geology or entomology where the research requires portable microscopic imaging device to be able to carry to field.

**Table 2:** Comparison of smartphone based imaging devices for vivid biomedical applications

Detection method	Target	Analytes/ technology used	Time for single test	Cellphone	Reference
Fluorescence and brightfield imaging	Blood cells, hemoglobin	Nucleic acid staining dye and custom build imaging cytometry platforms	10 seconds	Samsung Galaxy SII	[6]
Membrane technology with bio-chemiluminescence assay	Total bile acids and total cholesterol in whole blood	3α-hydroxylsteroid dehydrogenase and luminol−H_2_O_2_−horseradish peroxidase	3 minutes	iPhone 5S	[24]
Fluorescent imaging	*Cronobacter* spp.	Peptide nucleic acid (PNA) probes	-	Microsoft Lumia	[30]
Mie scattering detection, paper-based microfluidics	*Salmonella*	Antibody conjugated polystyrene submicroparticles	1 minutes	iPhone 4	[39]
Darkfield imaging with microfluidics	Bacteria and bacteriophage lysis	Melt-extruded fluoropolymer capillaries	4 hours	iPhone 6s/ Xperia L1	[40]
Colorimetric assays	Peanut allergen in food samples	ELISA allergen test kit, cellphone attachment with 2 test tubes and 2 LEDs	~20 minutes	Samsung Galaxy S II	[63]
Fluorescence-based lateral flow immunoassay	Avian Influenza (AI) virus strains	Nitrocellulose strip coated with anti-influenza antibody	15 minutes	Samsung Galaxy S3	[46]
Colorimetric assay with microfluidic device	BDE-47 in food sample	(L)-glutamate dehydrogenase (GDH), Diaphorase, Thiazolyl blue tetrazolium bromide (MTT)	15 minutes	___	[79]
Paper microfluidics with colorimetric assay	Red wine properties	Chemical dyes	___	iPhone 4	[80]
SPE and fluorescence spectroscopy	Antibiotic residues in milk	Photography lightbox with fluorescent light	___	iPhone 5s	[81]
Paper-based colorimetric assay	Glutamate in food compound, instant soup and wines	Glutamate-specific enzyme	___	Samsung Galaxy S3	[82]
Competitive immunoassay strip	OA and STX in shellfish	3D-printed smartphone strip adapter	30 minutes	iPhone 5s	[83]
Colorimetric imaging	Fluoride in water	Compact sample chamber adapter for Smartphone	___	Asus Zenfone, Moto G, Samsung DUOS	[84]
Disposable lateral flow-through strip	ALP as an indicator of incorrect milk pasteurization	pH indicators and phenylboronic acid	10 minutes	Vivo S7i	[85]
Brightfield imaging	Blood cells	Fiber-optic array, LED	<1 minutes	Samsung Galaxy S II, Sony-Ericsson	[86]
Fluorescence and brightfield imaging	*Plasmodium falciparum* and blood cells	455 nm LED, 20X wide-field microscope eyepiece, 100 nm fluorescent beads	2 minutes	Nokia N73	[87]

The development of smartphone-based imaging technologies has seen an exponential surge for the past decade now. With smartphones becoming an essential tool in everyday life, their hardware has improved a lot since the start of smartphone tales in the 1990s. Currently, smartphones are available at a much lesser price with better processing capability. Smartphone display and cameras are rapidly improving each year in terms of quality and sensor sensitivity respectively. The compact and easy handling characteristics makes it to be carried to the most remote places in the world. These will help in increasing the capacity of SIDs further. With the further incorporation of modern sensor in smartphone such as LIDAR or infrared (IR), they might find possible use in biomedical field in future. The only downside of these devices is the limited sensitivity of smartphone camera, which is not yet comparable to that of CCD or PMT. Furthermore, scientific cameras used in optical microscopes have a larger sensor size, enabling to capture more light to provide a better dynamic range and reduce the background noise of the images, whereas the smartphone camera sensors are kept small to fit the limited space in the device and are seen as the drawback limiting resolution, light intensity, or color balance in smartphone-based imaging devices. Also, the built-in image processing in a smartphone occurs after an image is captured, which changes the raw characteristics of the image causing variation in image properties and increases the risk of disease misdiagnosis [89]. Thus, SIDs are integrated with a deep learning algorithm trained with images from scientific cameras so that it can acquire similar image using the smartphone. This makes the whole process of image acquisition and analysis much faster and easier.

Such devices though showing better compatibility to the traditional device, their use in people’s everyday life is still uncertain. A large number of patents and publication results every year discussing various technologies coupled to smartphones, but researchers show less interest in translating their research into real-time products. Also, there exists a concern, such as smartphone-based technologies are over estimated and they do not add significant diagnostic or scientific value. However, the devices promises to transform lives if successfully integrated to existing smartphones. Thus, critical evaluation of their analytical performance is required to determine their performance as POC devices in resource-limited settings.
